# A Hybrid Lab-on-a-Chip Injector System for Autonomous Carbofuran Screening

**DOI:** 10.3390/s19245579

**Published:** 2019-12-17

**Authors:** Aristeidis S. Tsagkaris, Jana Pulkrabova, Jana Hajslova, Daniel Filippini

**Affiliations:** 1 Department of Food Analysis and Nutrition, Faculty of Food and Biochemical Technology, University of Chemistry and Technology Prague, Technická 5, 6–Dejvice, 166 28 Prague, Czech Republic; 2 Optical Devices Laboratory, Department of Physics, Chemistry and Biology—IFM, Linköping University, S-58183 Linköping, Sweden

**Keywords:** lab-on-a-chip, 3D-printed devices, microfluidics, food safety, pesticides, acetylcholinesterase, screening method

## Abstract

Securing food safety standards is crucial to protect the population from health-threatening food contaminants. In the case of pesticide residues, reference procedures typically find less than 1% of tested samples being contaminated, thus indicating the necessity for new tools able to support smart and affordable prescreening. Here, we introduce a hybrid paper–lab-on-a-chip platform, which integrates on-demand injectors to perform multiple step protocols in a single disposable device. Simultaneous detection of enzymatic color response in sample and reference cells, using a regular smartphone, enabled semiquantitative detection of carbofuran, a neurotoxic and EU-banned carbamate pesticide, in a wide concentration range. The resulting evaluation procedure is generic and allows the rejection of spurious measurements based on their dynamic responses, and was effectively applied for the binary detection of carbofuran in apple extracts.

## 1. Introduction

Food safety plays a crucial role in detecting the presence of harmful substances, such as pesticide residues, which can severely harm consumers’ health. Regulatory control of random samples, as commonly implemented in laboratories, yields less than 1% positive detections [1]. Thus, there is a demand for a more comprehensive complementary screening in order to protect the population from uncounted threats. In contrast to instrumental methods, which are expensive and time-consuming, simpler and more affordable screening tools are necessary. Here, we investigate such a possibility with a hybrid lab-on-a-chip multiple injector disposable device and processing methodology. The platform is able to perform the complete protocol necessary for a robust detection with smartphone cameras, within predicted semiquantitative categories and with the ability to reject failed measurements.

Carbamate (CM) and organophosphate (OP) insecticides in foods are globally used to boost agricultural productivity, and thus constitute a relevant target for in situ screening of samples. Although CMs and OPs do not usually release toxic metabolites and are degradable under ambient conditions, they feature neurotoxic effects originating from acetylcholinesterase (AChE) inhibition, a vital enzyme in the neural system of many organisms. AChE inhibition results in acetylcholine (ACh) accumulation, which can lead to serious health problems, including respiratory and myocardial malfunctions [2]. One such CM, carbofuran, is a broad-spectrum systemic insecticide, nematicide, and acaricide commonly used worldwide [3]. Although carbofuran has been reported as a highly harmful compound [4] and is banned in the European Union (EU), there are still carbofuran violation notifications in the EU Rapid Alert System for Food And Feed (RASFF, https://goo.gl/h1Hc41, last accessed on 13 September 2019), mostly from imported foodstuffs. Maximum residue limits (MRLs) have been set in various food matrices, for instance 0.001 mg/kg in apple, which is ten times less than the EU default MRL (0.010 mg kg^−1^), indicating harmful carbofuran potential.

Liquid and gas chromatography (LC and GC) coupled to tandem mass spectrometry (MS/MS) are the reference methods used for pesticide residues detection in foodstuffs [5,6]. Despite being highly accurate, robust, and with low limits of detection (LODs) at the sub-ppb level, chromatographic methods are also expensive, time-consuming, and require highly skilled operators. Therefore, such reference methods do not fit into the affordable complementary screening concept [7,8]. Alternative methods have been investigated, including immunoassays against specific CMs [9], electrochemical detection [10,11], surface plasmon resonance (SPR), and fluorescence aided by nanomaterials (NMs) [12]. Although these alternatives attain competitive LODs, they also suffer from high cost, complicated procedures, shelf-life limitations, and can require complex instrumentation [2,9,10,11,12]. 

On the contrary, paper-based assays fulfil many of the practical requirements for in situ detection. The technology is not only portable, easy to use, cost-effective, disposable, and available worldwide, but can also be functionalized with enzymes for affordable detection [13]. On the other hand, the integration of multiple-step protocols, which are well developed for classical lab-on-a-chip (LOC) methods, are not simple with paper devices. The challenge in this case was to enable the capabilities of active injectors in a disposable device integrating volume-metered reagents and references, as well as the paper detection stage.

AChE in vitro can hydrolyze certain substrates to colored products, and in the presence of an inhibitor its activity is reduced (producing less color). Although such sensors have been used for the detection of CMs and OPs, they were purely qualitative [14], relied on inaccurate visual inspection of the results [15,16], and lacked sample handling integration. 

In this study, semiquantitative, smartphone-based carbofuran screening was achieved using a hybrid 3D-printed paper–lab-on-a-chip injector device. Active injection in disposable devices is unusual due to the costs and complexity of micro injectors capable of delivering well-defined volumes [17]. The present work reports on a disposable device featuring four integrated injectors able to deliver 15 μL volumes on demand at freely configurable protocol stages. This capability is achieved by an asymmetric flow resistance provided by the functionalized AChE paper assay itself, acting as a permeable barrier in the LOC micro channels. The devices were measured in a 3D-printed holder under controlled illumination provided by a smartphone camera flash, while the phone rear side camera video recorded the dynamic color response of sample and reference carbofuran concentrations. Recording the dynamic response enabled the actual analyte effect to be disentangled from the wicking, incubation, and fluidic effects in coloration. The observed responses can be explained by a double exponential model, which was used to predict semiquantitative ranges for robust identification and rejection of suboptimal detections, while providing a generic procedure able to accommodate the binary detection of carbofuran in apple extracts.

## 2. Materials and Methods

### 2.1. Design and Fabrication

#### 2.1.1. Unibody-LOC (ULOC) Device

The ULOC devices were designed using Autodesk Inventor Fusion for Mac, and the generated .stl files were printed with a stereolithography (SLA) 3D printer (Formlabs, Form 1+) controlled by the PreForm 1.9 software, using Clear Type O4 resin and printing at 100 μm layer resolution.

ULOC devices were designed to self-support their features, thus avoiding the extra cost and time of creating supporting structures. For the current total volume of 2.28 mL, the cost of each prototype was 0.3€. Additionally, ULOC-implemented channels or chamber architectures open on at least one side, thus allowing easy removal of uncured resin by sonication in ethanol. The surfaces of the devices were refined using sandpaper, which allows the open channels to be sealed with adhesive tape. This step is no longer necessary with the next generation of SLA printers. 

In the case of the current device, Whatman paper functionalized for the colorimetric detection was fixed on the underside of the ULOC using a plastic spring element, which secured the contact of the paper with the end of the microfluidic channel, thus creating four porous injector barriers in each device. The top tape was trimmed to expose the cells to the ambient illumination in the detection zone, preventing any limitation to the flow and avoiding interferences to the optical path.

#### 2.1.2. Device Holder

The ULOC devices were measured within a holder that shielded ambient illumination and set a repeatable positioning with respect to the phone illumination (rear side flash) and camera. The holder was designed using Autodesk Inventor Fusion for Mac and printed with a fused deposition modeling (FDM) 3D printer (M3D) using a PLA filament. The design consists of 2 complementary parts used to attach the phone and guide the ULOC into position, and its final version was painted with black mat paint.

### 2.2. Chemicals and Reagents

Phosphate buffer saline (PBS) tablets, AChE from *Electrophorus electricus*, acetylthiocholine iodide (AThI, purity >99%), 5,5′-dithio bis-2-nitrobenzoic acid (DTNB, purity >99), bovine serum albumin (BSA), and Whatman® cellulose chromatography paper were from Sigma-Aldrich (Prague, Czech Republic). Anhydrous magnesium sulphate (MgSO_4_) was obtained from Fluka (Buchs, Germany), and sodium chloride (NaCl) from Penta (Chrudim, Czech Republic). Sorbent primary–secondary amine (PSA, Bondesil, 40 μm) was purchased from Agilent Technologies (USA). Deionized water was purified using a Milli-Q system (Millipore; Bedford, MA, USA). The 96-microwell plates were supplied by Gama Group (Ceske Budejovice, Czech Republic). AChE stock solution in PBS (2 U μL^−1^) was stored in the fridge at 4 °C. Acetonitrile were supplied as high purity solvents from Merck (Darmstadt, Germany). Carbofuran was analytical standard grade and purchased by Sigma Aldrich (Taufkirchen, Germany). Carbofuran stock solution (3.0 mg mL^−1^) was prepared in acetonitrile and kept in a freezer at −20 °C, protected from ambient light. Carbofuran working solutions were prepared daily in PBS. 

### 2.3. Enzyme Immobilization

AChE was physically adsorbed on Whatman paper strips with 1.4 × 1 cm dimensions. The immobilization procedure was simple, as 10 μL of AChE working solution in PBS (0.5 U μL^−1^) were pipetted into a microwell of a 96-microwell plate and a strip was dipped in the well using tweezers, resulting in a 5 U AChE/strip. Afterwards, the strips were dried at room temperature for 2 h and were ready for use. Measurements at this stage of proof of concept were conducted at room temperature, and the shelf-life of the paper assay was evaluated within a 56-day period (Figure S5), showing a stable behavior. 

### 2.4. Paper-Based AChE Assay

Immobilized AChE activity was measured both in the presence and absence of an inhibitor, carbofuran insecticide, using the Ellman’s assay, which is composed of two steps. Firstly, the paper strip was incubated with a sample for 8 min and then a solution of the enzyme substrate and the chromogenic reagent (75 mM AThI: 7.5 mM DTNB, 9:1 (*v*/*v*), respectively) was added, and the yellow color development was monitored for 3 mins using the 3D-printed smartphone reader.

### 2.5. Smartphone Readout

A Huawei P8 lite smartphone was used for the detection coupled to a 3D-printed holder. To achieve standardized and repeatable video acquisition, the free OpenCamera app was used, which permits the adjustment of exposure, focus, and illumination (see Supplementary Materials, Table S1). Videos of the whole enzymatic reaction were recorded at 30 fps (frames per second) and processed by Python software.

### 2.6. Python Software and Graphical User Interface (GUI)

Acquired videos were imported in Python, using a program with a tkinter user interface (Figure S6) that displayed an initial video frame to allow the interactive selection of coordinates for the regions of interest (ROIs), corresponding to the sample and reference cells. Besides the ROIs coordinates, the initial cut-off time (*to*) to eliminate the wicking stage and the total considered response time could be specified. Once such selection was complete, data evaluation consisted of the recording of the blue channel intensity for each frame averaged for all pixels of each ROI. Such response for the reference and sample cells were generated and saved in .csv format for further analysis.

A complementary Python program was developed to collect multiple .csv outputs and operate with such data to create response signals by subtracting the sample dynamic response from the reference response. Such signal was resampled to 5000 data points and curve fitted to a 6-degree polynomial function, to avoid overfitting when the signals were projected in the principal component (PC) space for categorization. The correct categories were produced by the model in the same PC space, which enabled semiquantitative validated ranges to be created that could be used for automatic validation or rejection of individual measurements. 

### 2.7. Food Samples 

Apple bio-samples were purchased by the local market. A representative portion was homogenized with a PM-100 Retsch Planetary Ball Mill (Haan, Germany) and the samples were kept at –20 °C. Prior to paper assay, the samples were analyzed with the liquid chromatography tandem mass spectrometry (LC-MS/MS) method (see Supplementary Materials) to verify that they did not contain detectable levels of CMs and OPs.

### 2.8. Food Samples Extraction

Quick, easy, cheap, efficient, rugged, and safe (QuEChERS) extraction is the golden standard for pesticide residue extraction, and thus it was applied to extract the tested samples as described in [18], with slight modifications. To begin with, 10 g of representative sample was weighted into a 50 mL solution. When needed, samples were spiked with target analytes from a freshly prepared solution (20 µg mL ^−1^) in acetonitrile to achieve the final concentration and left for at least 30 min at room temperature. Then, 10 mL of acetonitrile was added and the tubes were shaken vigorously for 2 min. To achieve phase separation, 4 g MgSO_4_ and 1 g sodium NaCl were added to each tube, shaken vigorously for 1 min, and finally centrifuged for 5 min at 10,000 rpm. Following the partitioning step, 3 mL of the acetonitrile extract was transferred to a 15 mL centrifuge tube containing 75 mg of PSA sorbent and 450 mg of MgSO_4_. The tube was shaken for 1 min and centrifuged for 2 min at 6000 rpm. Then, 500 µL of the cleaned-up extract was transferred to a vial and evaporated until nearly dry under a gentle nitrogen steam. Finally, samples were reconstituted to 500 μL of PBS and subjected to the enzyme paper assay.

## 3. Results and Discussion

### 3.1. Hybrid Injector Architecture and Function

To achieve a practical device configuration with potential for on-site use demands the integration of the paper assay into an autonomous LOC system able to support robust response evaluation [19]. Additionally, it is also imperative to conceive a completely disposable device compatible with the cost and user friendliness demands. Commonly, multiple step sample conditioning systems are forced to employ reusable parts, such as injectors, due to the normally elevated cost of such components. Here, we explored a design that allowed integration of the injectors at the same cost as the disposable LOC. For this purpose, the unibody-LOC (ULOC) concept was used. ULOC allows rapid prototyping of 3D-printed microfluidic devices and disposable optics using regular 3D printers reported elsewhere [20,21,22].

**Figure 1:**
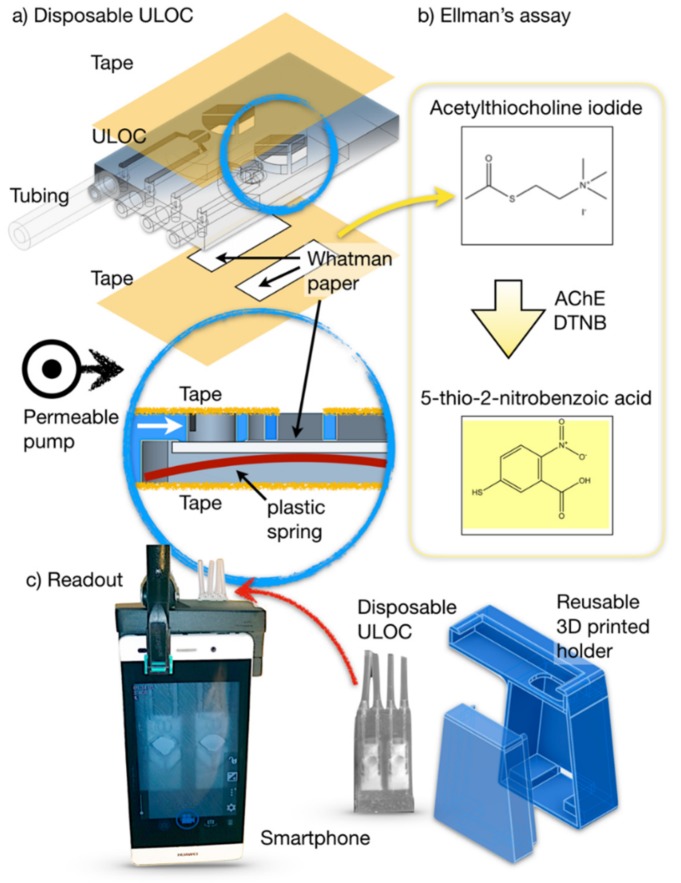
(**a**) Architecture of the unibody-LOC (ULOC) device with four injectors and the functional paper membrane containing the colorimetric assay. (**b**) Ellman’s assay detection principle implemented on the paper strip, which also acts as porous barrier for the hybrid injector. (**c**) A ULOC device in the holder with the screen showing the acquired image and a depiction of the device and the holder construction. Note: AChE = acetylcholinesterase; DTNB = 5,5′-dithio bis-2-nitrobenzoic acid.

ULOC can be configured to incorporate unidirectional valves and even zero dead volume check valve injectors at low cost [23]. However, it has never been used to connect classical fluidics with paper-based devices. Such a situation implies new synergies that can be used to simplify the architecture by introducing a new hybrid paper–ULOC type of injector. ULOC injectors require an asymmetric flow resistance for the forward and backward flow directions, which in previous cases has been created by an elastic element [22], but in the current design the paper itself offers a permeable element to asymmetrically restrict the backward flow (Figure 1a). Thus, the elastic tubing containing the measured sample or reagent volume is delivered towards the functionalized paper when pressurized, but when the tubing is released, the atmospheric pressure in the distal side is not enough to send the flow backwards, thus creating an effective forward flow for each activation of the injection tubing. Once the injected volume reaches the paper membrane, wicking of the paper membrane also contributes to the transport, such as in other microfluidic concepts [19,24].

The developed protocol (Figure 1b) requires AChE immobilization on paper strips (5 U AChE/strip, see Materials and Methods), assembly in the ULOC device, and finally injection of a 15 uL sample. In parallel with this operation, a reference concentration (blank) sample is measured in the control channel. Both reagent and reference concentrations are integrated in the injectors, and only the sample is pipetted in the detection cell injector, enabling a simple handling procedure.

The analytical measurement of colorimetric assays requires control of the image acquisition conditions, which in this case entails a device holder designed for the Huawei P8 lite. The design contemplates the alignment of the illumination provided by the phone flash and the phone camera to secure the most homogenous distribution of illumination on the reference and detection cells, without any further intensity compensation. The developed accessory also blocks ambient light and provides repeatable positioning of the measuring device (Figure 1c). The device response is captured in video at 30 frames per second (fps) using the free OpenCamera application, which enables continuous flash illumination, fixed focus, and constant exposure along the whole acquisition.

### 3.2. Data Acquisition

The measurement of a reference cell and a sample cell allows separation of the actual chemical response from changes in intensity due to the wicking process or the food matrix presence. The response of both cells is simultaneously recorded under the same illumination and the acquired video is analyzed with Python software. Such a program (Figure S6) allows the video to be loaded and introduces the coordinates of two ROIs, one for the reference cell and one for the sample cell, which are used to extract the dynamic response of the sample.

**Figure 2:**
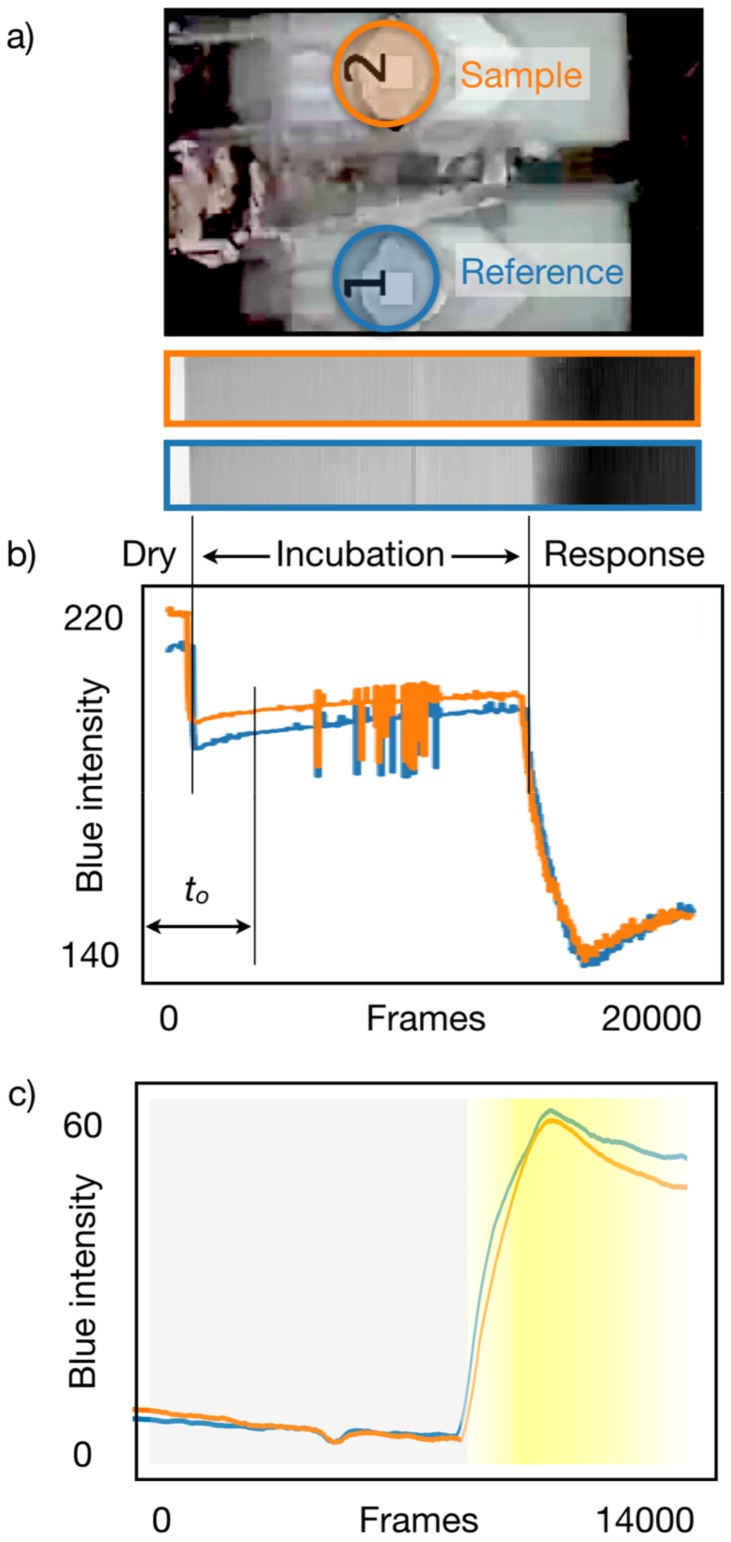
Data processing illustration of an acquired video: (**a**) one frame of the video acquisition showing the identification of regions of interest (ROIs) and the collection of the blue camera intensity across 20,000 frames acquired at 30 fps; (**b**) average intensity signal of each ROI, the discounted *to* data, and indication of incubation time; (**c**) aligned signals indicating the response (yellow hue).

Ellman’s assay displays its response as yellow hues, the variation of which is best captured in the blue channel of the video stream. Figure 2a shows the collection of both ROIs for all the frames of the captured video. This approach provides a complete recording, not only of the response in one instant, for example as in a classical end-point measurement, but also as a dynamic response that can be compared with a model to determine if a measurement was valid and performed in acceptable conditions.

The intensity in the blue camera channel for all pixels in each ROI is averaged for each frame and constitutes the raw signal displayed in Figure 2b. As reported in Figure S3, the reproducibility of the measuring platform is within one-pixel count, and it is achieved by proper design of the illumination and detection, without any sample treatment. The reflectance spectroscopy of the assay on paper (Figure S2) indicates that all valid responses occur within the blue spectral window of the camera, thus excluding any blending of color channels as a possible alternative. This result indicates that the total systems variability originates in the chemistry and sample delivery, which are the areas to improve in future efforts.

When the first reagent is introduced and wets the paper, there is a decrease in the blue channel intensity, and when the analyte is injected, after a determined incubation time, the color response starts building up, showing a further decrease in the blue intensity. Figure 2b corresponds to the same concentration of analyte applied to both sample and reference cells, and is used to determine the best achievable equivalence between both cells within four intensity counts.

Another aspect that can be observed is the different delay between the injection of samples to each cell, and later the injection of the reagent. These delays are automatically identified, as well as the onset of the response to allow alignment of the reference and detection responses for the characterization, categorization, and semiquantification.

Since the test is run with a defined protocol, the easiest way to eliminate the transition between dry paper and wetting is to use a fixed time to remove this initial transition (*to*), whereas to identify the time of the response onset, the frame of the minimal derivative of the reference signal is used. The shift between responses is then subtracted, resulting in the alignment of both responses. After removing the data before *to*, the maximum value corresponds to the baseline with wet paper, and thus the amplitudes can be aligned as well (Figure 2c).

Finally, the response signal is computed as the difference between the sample and reference cell responses, thus providing a unified signal disentangled from the color variation due to wicking and fluidics transport, which highlights the actual response to the target analyte. 

### 3.3. Semiquantification Using a PCA Acceptance/Rejection System 

**Figure 3:**
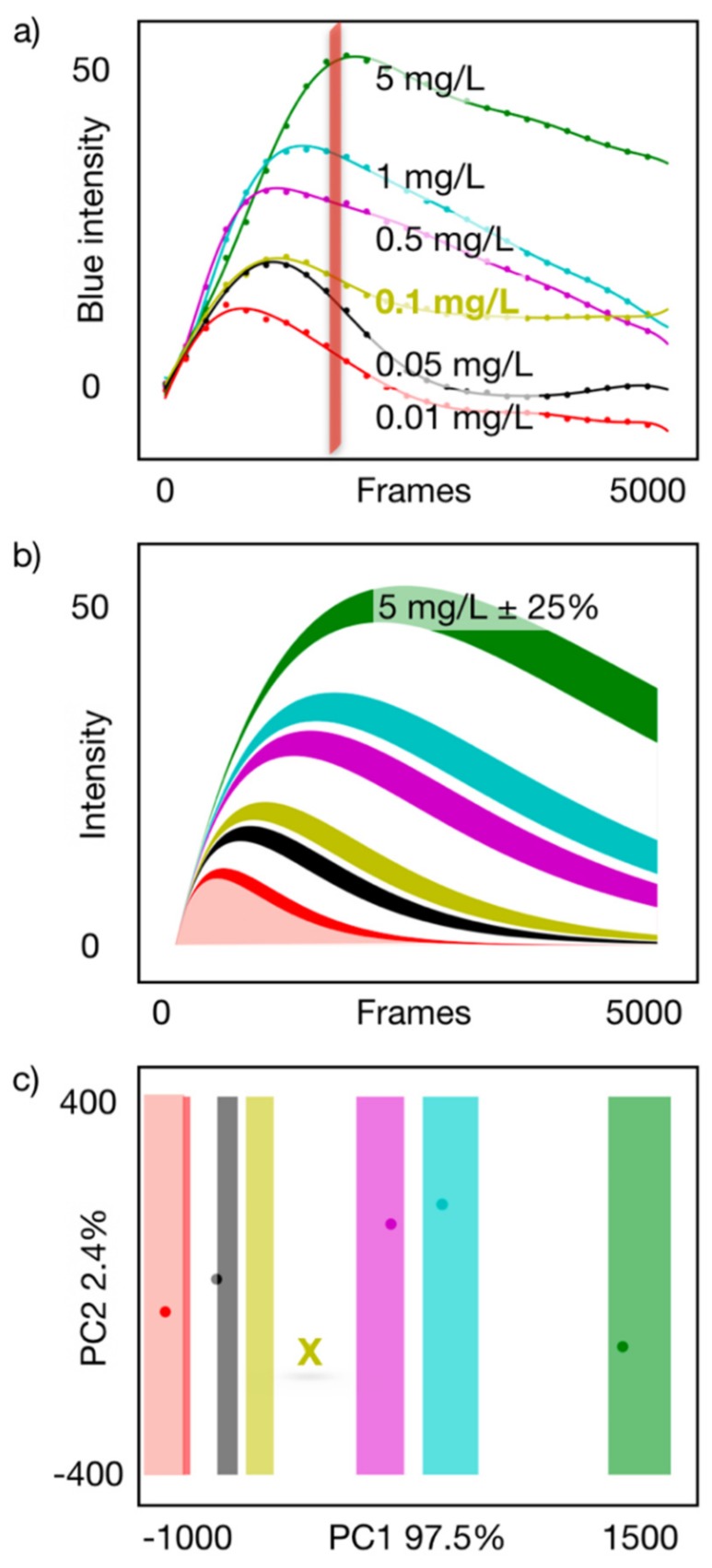
(**a**) Curve fit responses to concentrations between 0.010 mg L^−1^ to 5.0 mg L^−1^ of carbofuran in phosphate buffer saline (PBS); (**b**) model representing the observed response behavior and acceptance band for each semiquantitative category; (**c**) projection of acceptance bands and experimental response showing automatic categorization ranges and rejection of a measurement outside valid ranges (0.100 mg L^−1^ in this case). Note: In all cases, green font corresponds to 5.0 mg L^−1^, blue font corresponds to 1.0 mg L^−1^, magenta font corresponds to 0.500 mg L^−1^, yellow font corresponds to 0.100 mg L^−1^, black font corresponds to 0.050 mg L^−1^ and red font corresponds to 0.010 mg L^−1^. PC1 = first principal component; PC2 = second principal component.

Figure 3a collects the dynamic responses for different concentrations of carbofuran after the subtraction of the reference. Since the effect of the analyte is the inhibition of the color response, it follows that lower concentrations correspond to faster responses (Figure 3a), and the complete dynamic range between 0.010 mg L^−1^ to 5.0 mg L^−1^ can be covered within 3 minutes (the number of frames correspond to a 30 fps acquisition, accordingly 5000 frames equals 2.77 min).

The observed behavior can be explained by a double exponential function, with one of the time constants modulated by the analyte concentration.

$$s\left(t\right)=A\times \left(1-e^{\frac{-t}{\tau_{1}}}\right)\times e^{\frac{-t*c^{\gamma }}{\tau_{2}}}.$$

where *s(t)* is the response to the analyte *A*; τ_1_, τ_2_ are the signal amplitude and time constants, respectively. The expression explains the shape of the whole set of concentrations, *c*, and γ = 0.3 is the single parameter that adjusts the relation of all response amplitudes. The current equation is an ad hoc function aiming at describing the system behavior for the full range of concentrations with few fitting parameters, and to predict the shape of correct responses for result validation. The use of an equation for image processing was also reported by Lai et al. [25].

The modeled concentration ranges enable semiquantitative acceptance bands to be defined (Figure 3b), which translate into classification ranges in a principal component analysis (PCA) representation (Figure 3c), providing a criterion to accept only valid responses. A simple end-point evaluation could be feasible for this assay, however, a spurious measurement (yellow example) could pass as a valid result, whereas the current system dismisses it. Thus, the advantage of the video recording is to transform the shape of the response into an indicator of valid experiments, which when projected into a principal component space, enables automatic categorization of such concentrations and rejection of spurious measurements (Figure 3b). In addition to the rejection/acceptance band, each semiquantitative categorization score has its own range of validity, accommodating the nonlinearity of the response along the entire dynamic range and negotiating the variability of each quantitative category.

### 3.4. Binary Carbofuran Screening in Apple Extracts

**Figure 4:**
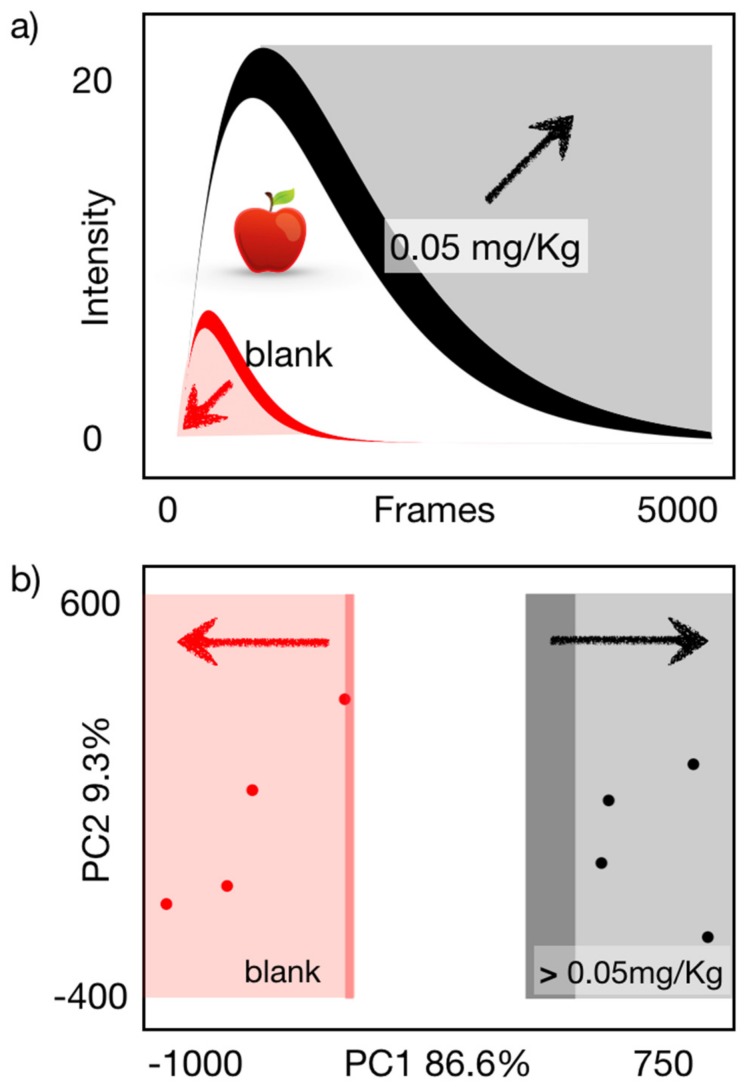
(**a**) Model representing the observed response behavior and acceptance band for the binary discrimination of carbofuran in apple extracts. (**b**) Principal component analysis (PCA) scores for blank (n = 4) and contaminated apple extracts (n = 4), indicating the effective separation between the two groups.

To demonstrate the applicability of the developed analytical platform in food matrices, apple samples were screened for carbofuran at a concentration level of 0.050 mg Kg ^−1^. This concentration was selected based on the calibration curve results as a compromise between reliable (avoiding false negative results) and sensitive screening. In this way, four blank apple samples and four spiked apple samples (final concentration was 0.050 mg Kg ^−1^) were successfully discriminated using the described approach (Figure 4). These results were also verified using an accredited LC-MS/MS method (see Supplementary Materials). Although it was not feasible to achieve LODs lower than EU MRLs (0.001 mg Kg^−1^ for apple), the platform is suitable to control the EU acute reference dose (ARfD), which can be exceeded under certain consumption conditions. Importantly, the attained performance improves the detection limit of colorimetric methods reported for other AChE inhibitors by between 4 times and 2 orders of magnitude [14,26,27]. Compared to chemiluminescent methods, which have been also used as screening tools, Liu et al. [28] developed a paper-based chemiluminescence chip for dichlorvos monitoring in vegetables, detecting the analyte at ppm level, which is significantly higher than the developed method screening concentration. In another study [29], sensitive detection of chlorpyrifos was achieved in spinach samples (LOD = 0.5 × 10^−7^ M or 0.017 mg Kg^−1^) using molecular imprinting (MIP) coupled to chemiluminescence detection. However, although MIP enhanced sensitivity, there was no portability potential. Consequently, the proposed concept paves the road towards in situ detection of pesticide residues, and at the current stage can be useful for complementary low-cost screening of imports from countries where carbofuran use is still permitted.

## 4. Conclusions

The current concept provides an autonomous platform to exploit the benefits of low-cost paper assays by complementing its weaknesses with tailormade disposable microfluidics, thus delivering on demand reagent and sample injection for a multiple-step protocol (0.30 €/device at the development stage). It must be noted that the reported cost of 0.30 €/device is a highly affordable development cost, which is made possible by modern 3D printing technology. This cost enables multiple design iterations a day with any number of modifications. In this particular case, more than 14 different optimization cycles were conducted during a 10 day period, with the ability to test each physical outcome. The natural next step is to migrate the design to scale manufacturing (e.g., injection molding) to make the final cost compatible with the paper fluidics. 

Importantly, the collateral use of the paper assay as a porous barrier enabled the simplification of the LOC architecture, and allowed the integration of a reference channel at the same cost. The combination of the autonomous device with a proper light- and exposure-controlled acquisition extended the possibilities of the colorimetric assay to adopt a robust semiquantitative configuration, which enabled the differentiation of valid response identification from spurious measurements. The proposed concept provides a screening procedure for the binary identification of carbofuran in apple matrix and could support screening of different CMs and OPs at different concentration levels, since they share a similar inhibitory effect against AChE.

## Supplementary Materials

The following are available online at https://www.mdpi.com/1424-8220/19/24/5579/s1, Figure S1: Carbofuran calibration curve in PBS (n = 9 for each calibration point), Figure S2: Kinetic determination of a blank sample reflectance intensity, Figure S3: Detected color intensity of artificial yellow color defining the minimum attainable device error and the correct position of the Huawei P8 smartphone for all measurements, Figure S4: Dynamic color development using AChE concentrations of 1, 2, 5, and 10 U/strip, Figure S5: Color intensity of AChE strips prepared using physical adsorption principle during a 56-day period, Figure S6: G graphical user interface (GUI) for video analysis, Figure S7: Different carbofuran concentrations as measured with a LC-MS/MS system, Table S1: Camera settings during smartphone colorimetric detection, Table S2: Carbofuran detection in apple using liquid chromatography coupled with tandem mass spectrometry (LC-MS/MS).
