# Supplementary material for: A Hybrid Lab-on-a-Chip Injector System for Autonomous Carbofuran Screening
Source: Sensors (Basel). 2019 Dec 17;19(24):5579. doi: 10.3390/s19245579 (PMC6960838; doi:10.3390/s19245579)
Supplement: Supplementary file 1 [file sensors-19-05579-s001.pdf]

SUPPLEMENTARY MATERIAL

A Hybrid Lab-on-a-Chip Injector System for Autonomous Carbofuran Screening

Aristeidis S. Tsagkaris[1,] *, Jana Pulkrabova[1], Jana Hajslova[1] and Daniel Filippini[2,] *

[1] Department of Food Analysis and Nutrition, Faculty of Food and Biochemical Technology, University of Chemistry and Technology Prague, Technická 5, 166 28 Prague 6 – Dejvice, Prague, Czech Republic

[2] Optical Devices Laboratory, Department of Physics, Chemistry and Biology—IFM, Linköping University, S-58183 Linköping, Sweden.

* Correspondence: daniel.filippini@liu.se (DF) and tsagkara@vscht.cz (AT)

1. **Carbofuran inhibitory effect against AChE using a microplate assay**

Here, 30 μL AChE (0.009 U well^{-1}) was incubated with 30 μL of an inhibitor for 15 min. Next, 30 μL of a 12.5 mM AThI to 1.5 mM DTNB (9:1) solution in PBS were added and the absorbance was measured at 412 nm after 2 min.

The experiments were performed on three independent days, in triplicate, and the data were pooled (Figure S1, n = 9 for each calibration point). To obtain the 20% values of the maximal and half maximal inhibitory concentration (IC_{20} and IC_{50}, respectively), a pesticide was prepared at seven different concentration levels (Table S1) and compared to a control sample. Dose–response with variable slope analysis was performed using GraphPad prism 5.0 software.

IC_{20} and IC_{50} are important characteristics of an inhibition assay, indicating its detection capability. Thus, the optimized AChE assay was performed to find the inhibitory potency of carbofuran in a PBS. Carbofuran solutions were prepared in the range 0.035–2.2 μM or 0.008-0.5 mg/L (n = 7 calibration points and one blank sample). An IC_{20} equal to 0.037 μM (0.033–0.041 μM) or 0.008 mg L^{-1} (0.007–0.009 mg L^{-1}) was found, while IC_{50} was 0.099 μM (0.092–0.11 μM) or 0.022 mg L^{-1} (0.020 – 0.024 mg L^{-1}).

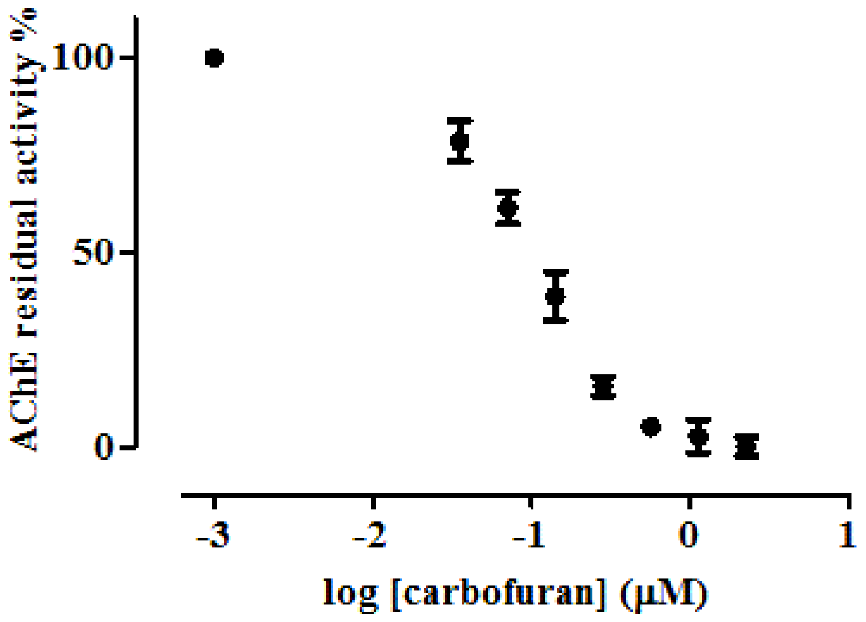

Figure S1. Carbofuran calibration curve in PBS (n = 9 for each calibration point).

2. **Spectral characterization of the enzyme strips**

The reflectance intensity of the paper-based AChE assay was investigated by kinetically testing a blank sample (Figure S2). It was revealed that that the response mostly modulates the blue region of the spectrum, whereas the red band has a flat reflectance. Measurements were taken using an Ocean Optics 2000 USB optical fiber spectrophotometer equipped for spectral reflectance measurements.

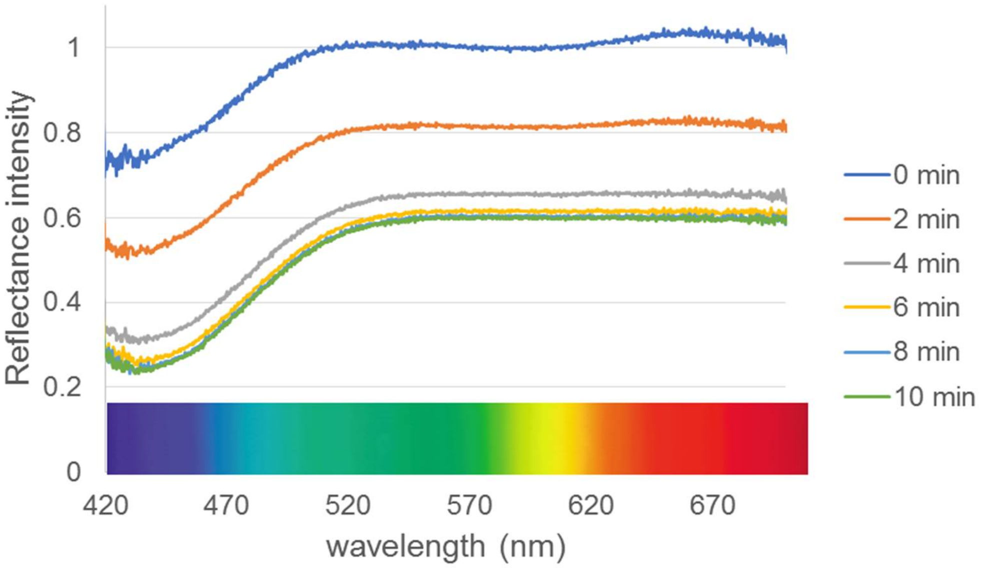

Figure S2. Kinetic determination of a blank sample reflectance intensity.

3. **Open camera settings**

Table S1. Camera settings during smartphone colorimetric detection.

Settings	
**Video frame rate**	30 fps
Camera flash	Constantly on
ISO value	100
Exposure	Locked
Exposure compensation	+1EV
Zoom	X 1.1

AChE strip dimensions and illumination

The developed assay is based on colorimetric detection, meaning that the measurements are vulnerable to positioning of the illumination and the camera, which can be optimized for every smartphone model. To characterize the positioning, a yellow food colorant was used to mimic the enzymatic reaction, and paper strips were cut to 1.4 cm × 0.5 cm. At the best positioning, the two chambers could provide identical results (Figure S3), and this configuration was used in all experiments. As is depicted, the difference is smaller than in the real assay implementation (Figure

2c), in which case the difference can be entirely attributed to the variability of the chemistry implemented in the assay.

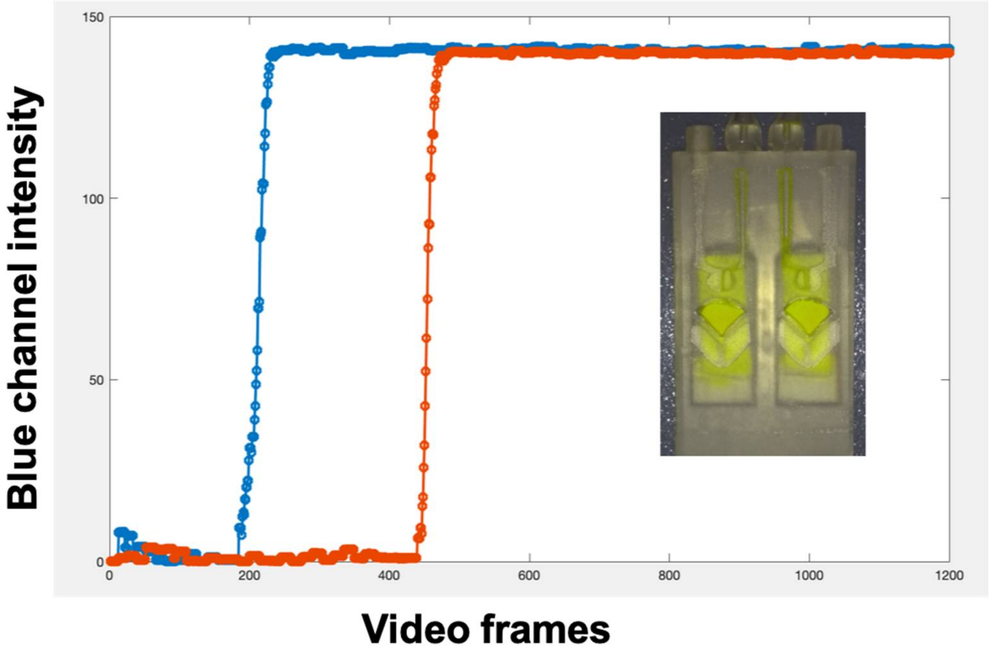

Figure S3. Detected color intensity of artificial yellow color defining the minimum attainable device error and the correct position of the Huawei P8 smartphone for all measurements.

4. **Effect of AChE concentration on color intensity**

Color cameras in smartphones are equipped with 8-bit intensity resolution for the red, green, and blue channels, meaning that a single acquisition can be resolved at 1/255 in the best case, if the color signal spans the complete 0–255 range of a channel. Four different AChE concentrations were tested (1 U/strip, 2 U/strip, 5 U/strip, and 10 U/strip) in triplicate to optimize assay conditions. The increase in AChE concentration resulted in larger color intensity (Figure S4). The more enzymes that are present, the faster the response, but there was no gain beyond 5 U/strip, which is the best compromise of color range, response speed, and reagent economy.

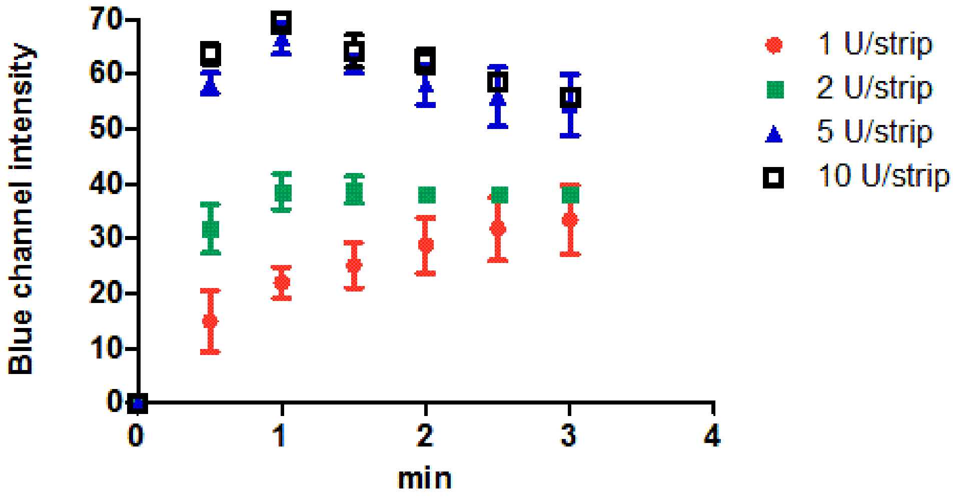

Figure S4. Dynamic color development using AChE concentrations of 1, 2, 5, and 10 U/strip.

5. **Sample volume**

Sample volume and the associate volume of reagents and reference affect both the assay detection capability and magnitude of color development response. The sample volume reflects the extract amount that will be needed to perform the test in a food matrix. Thus, 10 and 15 μL PBS (n=2 per volume) were tested using AChE strips (5 U/strip). Although the response was fast for all the volumes, 10 μL were insufficient to provide homogenous wetting of the AChE strip and 15 μL resulted in a good compromise that minimized sample volume and reagent consumption. Therefore, 15 μL sample volume was adapted for the experiments.

6. **Shelf-life**

Paper assay shelf-life is indispensable for demonstrating the practical applicability of a screening test. For this reason, a batch of AChE strips was prepared the same day using the physical adsorption principle. The strip dimensions were 1.4 cm × 1 cm, while the concentration of the immobilized enzyme was 5 U/strip. Blank measurements were performed in triplicate (n=3) right after the strip preparation (day 0) and at specified time slots (Figure S5). During this 56-day period, the prepared AChE strips were packed in foil and stored in the fridge (4 °C). It was found that strips retained satisfactory activity for about two months.

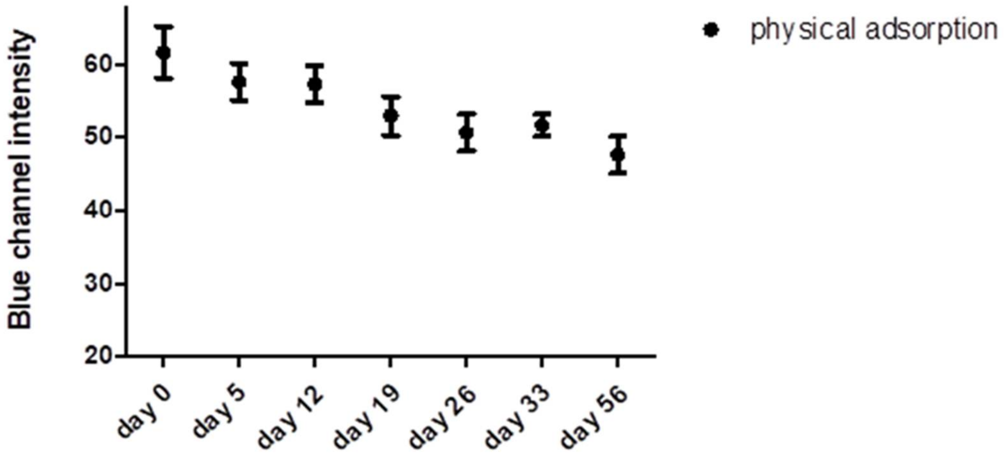

Figure S5. Color intensity of AChE strips prepared using physical adsorption principle during a 56-day period.

7. **Computer application/GUI**

A Python program with a graphical user interface (GUI) in tkinter was developed, which enabled recorded videos to be uploaded and the coordinates of regions of interest (ROIs) in the reference and sample cells to be interactively selected, as well as the cut off time. Once selected, the software computed the blue channel intensity responses of both cells, and stored them in .csv format for further processing.

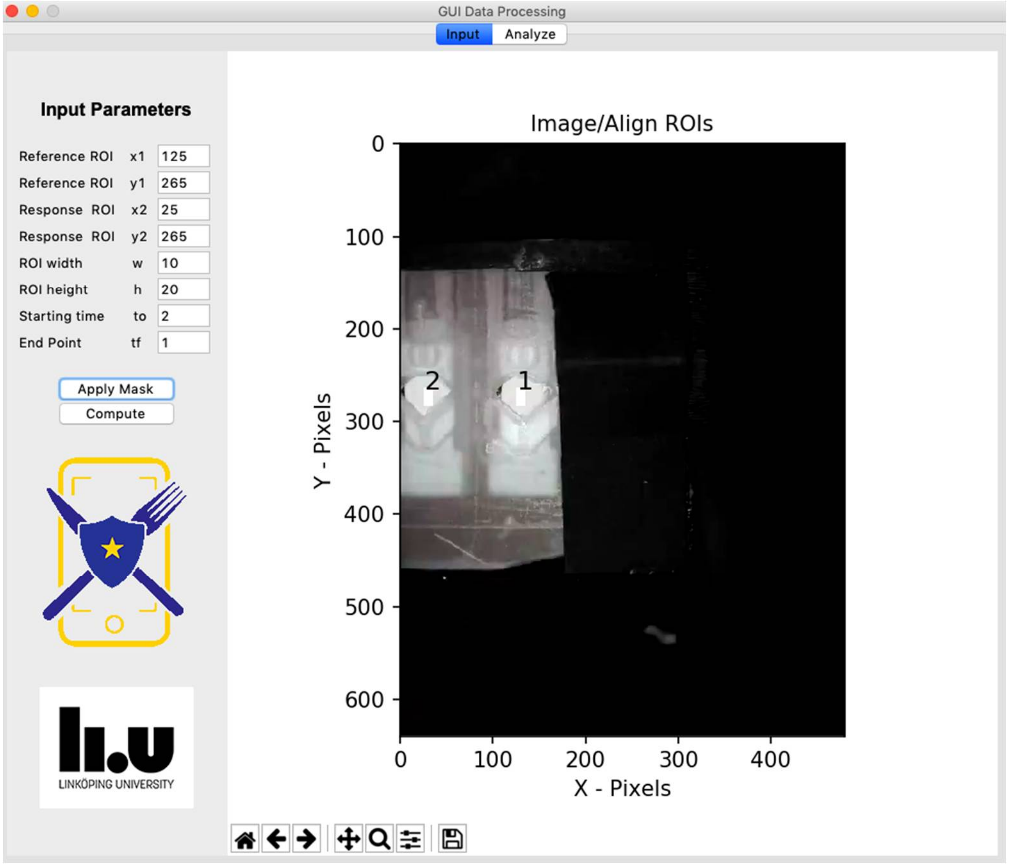

Figure S6. G graphical user interface (GUI) for video analysis.

8. **Liquid chromatography tandem mass spectrometry analysis**

A 1290 Infinity II liquid chromatography (LC) system (Agilent Technologies, Santa Clara, USA) coupled to a Triple Quadrupole G6495 (Agilent Technologies) mass spectrometer with electrospray ionization in positive ion mode (ESI+) was used for pesticide residue analysis. Dynamic multiple reaction monitoring (dMRM) mode was selected as the operation mode. The separation took place in an Acquity UPLC HSS T3 analytical column (100 mm x 2.1mm i.d. x 1.8 µm particle size, Waters, Milford Massachusetts, USA) with controlled temperature at 40 °C, using water and MeOH (with the addition of ammonium formate and formic acid) as a mobile phase and a gradient described in [25]. The obtained data were processed using MassHunter software version B.07.00.

9. **Benchmarking towards LC-MS/MS**

The results of a screening test need always to be confirmed by a confirmatory method. Therefore, apple samples were analyzed using an accredited liquid chromatography tandem mass spectrometry (LC-MS/MS) method. The chromatographic method results were in line with the paper-based assay (Table S2 and Figure S7).

Table S2. Carbofuran detection in apple using liquid chromatography coupled with tandem mass spectrometry (LC-MS/MS) system.

carbofuran concentration	sample	spiked (mg Kg^{-1})	found (mg Kg^{-1})
	a	0	not detected
	b	0	not detected
	c	0	not detected
blank	d	0	not detected

	a	0.050	0.049
	b	0.050	0.048
	c	0.050	0.049
0.050 mg/kg	d	0.050	0.051

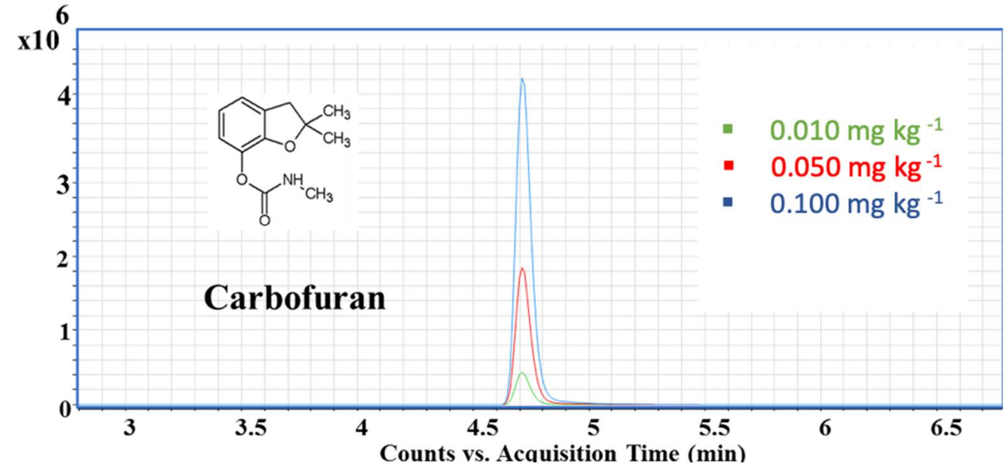

Figure S7. Different **c**arbofuran concentrations as measured with a LC-MS/MS system.
